# Metabolic alterations and cellular responses to β-Hydroxybutyrate treatment in breast cancer cells

**DOI:** 10.1186/s40170-024-00339-1

**Published:** 2024-05-29

**Authors:** Hadas Fulman-Levy, Raichel Cohen-Harazi, Bar Levi, Lital Argaev-Frenkel, Ifat Abramovich, Eyal Gottlieb, Sarah Hofmann, Igor Koman, Elimelech Nesher

**Affiliations:** 1 Department of Molecular Biology, Ariel University, Ariel, 4070000 Israel; 2 Institute for Personalized and Translational Medicine, Ariel University, Ariel, 4070000 Israel; 3 Rappaport Faculty of Medicine and Research Institute, Technion, Haifa, 3525422 Israel; 4 Medical Faculty Mannheim, Heidelberg University, 68167 Mannheim, Germany

**Keywords:** Ketone bodies, βHb, Beta-hydroxybutyrate, MCF7, Breast cancer

## Abstract

**Background:**

The ketogenic diet (KD), based on high fat (over 70% of daily calories), low carbohydrate, and adequate protein intake, has become popular due to its potential therapeutic benefits for several diseases including cancer. Under KD and starvation conditions, the lack of carbohydrates promotes the production of ketone bodies (KB) from fats by the liver as an alternative source of metabolic energy. KD and starvation may affect the metabolism in cancer cells, as well as tumor characteristics. The aim of this study is to evaluate the effect of KD conditions on a wide variety of aspects of breast cancer cells in vitro.

**Methods:**

Using two cancer and one non-cancer breast cell line, we evaluate the effect of β-hydroxybutyrate (βHb) treatment on cell growth, survival, proliferation, colony formation, and migration. We also assess the effect of KB on metabolic profile of the cells. Using RNAseq analysis, we elucidate the effect of βHb on the gene expression profile.

**Results:**

Significant effects were observed following treatment by βHb which include effects on viability, proliferation, and colony formation of MCF7 cells, and different effects on colony formation of MDA-MB-231 cells, with no such effects on non-cancer HB2 cells. We found no changes in glucose intake or lactate output following βHb treatment as measured by LC-MS, but an increase in reactive oxygen species (ROS) level was detected. RNAseq analysis demonstrated significant changes in genes involved in lipid metabolism, cancer, and oxidative phosphorylation.

**Conclusions:**

Based on our results, we conclude that differential response of cancer cell lines to βHb treatment, as alternative energy source or signal to alter lipid metabolism and oncogenicity, supports the need for a personalized approach to breast cancer patient treatment.

**Supplementary Information:**

The online version contains supplementary material available at 10.1186/s40170-024-00339-1.

## Background

Cancer cells are known to differ from normal cells in their metabolism. The utilization of metabolic pathways is altered in many ways such as pentose phosphate cycling, changes in energy utilization by glycolysis, increased metabolism of L-glutamine, as well as other shifts in the nutrients required for cell growth [1]. Increased glucose uptake and lactate production with a high rate of glycolysis and a low rate of oxidative phosphorylation, known as the Warburg effect [2], accelerates pathways required for rapid growth and proliferation while reducing the efficiency of energy production per unit of glucose [1]. At the same time, the Warburg effect is not universal and some tumors may corrupt neighboring stromal fibroblasts such that they process high levels of glucose, while the tumor cells themselves take up the biochemical products and use oxidative phosphorylation (the reverse Warburg effect) as their primary source of energy [3].

Since the metabolic program of cells is linked to the nutrients provided by dietary components, a strong association between cancer development and progression and diet has been described [4, 5]. Since the multiple subtypes of breast cancer are characterized by the expression of hormone receptors including estrogen receptor, progesterone receptor and human epidermal growth factor receptor 2 (HER2), the nutritional programs which modulate both nutrition and hormone balance have gained interest as potential adjuvant therapy specifically for breast cancer [6]. Recently, the ketogenic diet (KD) has been proposed for the treatment of certain types of cancer [7, 8]. The KD is based on high fat (over 70% of daily calories), low carbohydrate, and adequate protein intake [9]. Under ketogenic conditions, such as starvation, the levels of glucose, insulin, and insulin-like growth factors (IGF) [9] in the blood decrease and stabilize, and the liver produces ketone bodies (KB): acetone, acetoacetate (AcA) and β-hydroxybutyrate (βHb) by beta-oxidation of fatty acids, as an alternative energy source [10]. Therefore, the metabolic changes, resulting from either or both of these processes, have been predicted to have a beneficial effect in cancer treatment through a decrease in the blood glucose level, challenging the Warburg effect and limiting the available energy for cancer progression [9]. Still, the direct effect of KB on cancer cells remains unclear. Some studies demonstrate that treatment with AcA reduced cell growth [11], and viability [12], decreased ATP production [13], increased apoptosis in cancer cells [12], induced oxidative stress [14], diminished cancer cachexia, and altered metabolic profile [15]. Though some in vitro studies may be plagued by technical issues [16]. The more prevalent KB, βHb [17], in turn, delivers controversial results [18]. There is some evidence that βHb decreases cell viability [12, 19] and increases apoptosis [12, 15]. It has also been found to reduce oxidative stress, inflammation, cancer growth, and angiogenesis [20]. On the other hand, βHb may increase tumor growth [21], promote proliferation, migration, and stemness [22, 23]. Focusing on breast cancer, βHb shows no effect on cell proliferation [24], while it can serve as a fuel for mitochondrial activity of tumor cells, increasing their “stemness” and metastatic properties, that is further reflected in the correlation of poor clinical prognosis with high blood ketone levels [21, 25].

KBs are known to be elevated in the blood under both KD and starvation conditions [9]. Given the reduced preference for KB utilization when other nutrients are available, we conducted an investigation to assess the impact of βHb treatment in a medium that lacked alternative, more favored energy sources like glucose, pyruvate, and L-glutamine.

Due to the inconsistent results of KD conditions on cancer cell growth, survival, and progression reported in the literature, we hypothesized that KB may affect cancer cells in two different modes, simultaneously. On one hand, KB may affect tumor oncogenicity via signaling, thus influencing gene expression. On the other hand, KB may be used by cancer cells as an alternative source of energy in a state of nutrient deficiency. Both possibilities are dependent on the natural ability of the tumor cells to take up and to utilize KB. Thus, the primary aim of this study was to elucidate the effect of KBs on cancer cell metabolism and oncogenicity using a model of breast cancer under KD and starvation conditions.

## Materials and methods

### Cells & treatments

Breast cancer cell line MCF7 was received as a kind gift from Prof. Richard Feinman (SUNY Downstate Medical Center, NY). MDA-MB-231, another breast cancer cell line, was purchased from ATCC (Cat#CRM-HTB-26). Non-cancer HB2, a human mammary luminal epithelial cell line, was a kind gift from Prof. Ido Wolf (Tel Aviv Sourasky Medical Center, Tel Aviv, Israel). All cell lines were checked for mycoplasma infection on a monthly basis. All cells were cultured in high glucose (25mM) Dulbecco’s Modified Eagle’s medium (DMEM; Cat#01-052-1 A, Biological Industries) containing 10% fetal bovine serum (FBS; Cat#12657-029, Gibco), 2mM L-glutamine (Cat#03-020-1B, Biological Industries), 100 U/ml penicillin and 0.1 mg/ml streptomycin (Cat#03-031-1B, Biological Industries) at 37°C with 5% CO_2_. Cells were passed every 3–4 days using trypsin-EDTA (Cat#15,050,065, Gibco). Sodium β-hydroxybutyrate (βHb; Cat#54,965, Sigma-Aldrich) was prepared to a stock concentration of 800mM dissolved in ddH_2_O, filtered, aliquoted and stored at -20°C. Prior to experiments, βHb was diluted to working concentrations of 3 and 10mM in culture media.

In the experimental procedures, the cells were seeded in high glucose media and incubated overnight to allow for attachment. After 24 h, the medium was replaced with the appropriate medium according the experimental condition. “Ketogenic diet” conditions indicates low glucose DMEM (5.5mM; Cat#31,885,023, Gibco), and “starvation” conditions indicates DMEM with no glucose, no L-glutamine, no phenol red, and no sodium pyruvate (Cat#A14430-01, Gibco). Both contain 10% FBS and 100 U/ml penicillin and 0.1 mg/ml streptomycin, while only the low glucose media was also supplemented with 10mM L-glutamine (Cat#03-020-1B, Biological Industries). The treatment βHb was added at indicated working concentrations. Control groups were treated with low glucose media without βHb. The media in KD condition was replaced every 48 h, while in starvation condition the media was not changed in the middle of the experiments.

### Resazurin-based cytotoxicity assay

#### Cell viability under “ketogenic diet” conditions

Cells were seeded in 96-well plates, at 1.5 × 10^3^ cells/well and treated with 3 or 10mM βHb. These concentrations lie within the range observed in the context of starvation and a ketogenic diet. They were chosen because the physiological concentrations of βHb in serum can reach 6–8mM with prolonged starvation and above 2mM with a ketogenic diet [17, 26]. Following a total of 96 h of incubation, cell viability was analyzed using a cytotoxicity assay based on resazurin sodium salt (Cat#R7017, Sigma-Aldrich) solution in PBS. The 6 × (2.5mM) stock solution was applied to each well to a final concentration of 1x. After 4 h of incubation at 37^o^C, fluorescence was measured at 560_Ex_/590_Em_ nm using the ClarioStar plate reader (BMG LABTECH).

#### Cell viability under “starvation” conditions

Cells of different cell lines were seeded in high glucose media in 96-well plates, at concentrations to yield a confluence of 70% the next day. After 24 h, the medium was changed to DMEM no glucose, no L-glutamine, no phenol red, no sodium pyruvate (Cat#A14430-01, Gibco) containing 10% FBS (Cat#12657-029, Gibco) and 100 U/ml penicillin and 0.1 mg/ml streptomycin (Cat#03-031-1B, Biological Industries) with treatments. The media was supplemented with nutrients components, Sodium Pyruvate (Cat#03-042-1B, Biological Industry) 0.25, 0.5 and 1mM, or Glucose (Cat#A24940-01, Gibco) 1, 2.75, 5.5, 25mM, or L-glutamine (Cat#03-020-1B, Biological Industry) 1, 5, and 10mM. 10mM βHb was added to experimental groups, while the control groups were the same additional nutrient components without βHb. Following the total incubation time, cell viability was analyzed using a cytotoxicity assay based on resazurin sodium salt (Cat#R7017, Sigma-Aldrich) solution in PBS. The 6 × (2.5mM) stock solution was applied to each well to a final concentration of 1x. To achieve results, resazurin reagent was applied to each well on the 6th day of treatment and incubated for 24 h at 37^o^C, fluorescence was measured at 560_Ex_/590_Em_ using the ClarioStar plate reader (BMG LABTECH). All data is presented as the βHb OD score normalized to the appropriate non- βHb control, according to the following equation:

$$\eqalign{& percetage\,of\,BHb\,effect\, = \cr & \left( {\left( {{{OD\,of\,BHb\,treated\,cells} \over {Average\,OD\,of\,control\,group}}} \right)*100\,\% } \right)\, - \,100 \cr}$$

### Colony formation assay

One hundred cells were seeded per well in 48-well plates, with care taken to produce a single cell solution and avoid clumping and treated with 3mM or 10mM of βHb in KD media. The media was replaced every 48 h. Control groups were grown in the same media without βHb. When neighboring colonies reached each other, the experiment was terminated and colonies were stained with 0.5% methylene blue (Cat#M4159, Sigma-Aldrich), in 50% methanol (Cat#S93301, Thermo-Fisher) and manually counted. Results were analyzed by calculating the percentage of number of colonies formed by treated samples as compared to control.

### BrdU proliferation assay

Cells were seeded in 96-well plates, as 2 × 10^3^ cells/well and treated with 3 or 10mM βHb in KD media. BrdU proliferation assay (Cat#QIA58. Sigma-Aldrich) was performed after 96 h of incubation. BrdU 1000x was added to a concentration of 1x. The plate was incubated for 4 h. The level of BrdU was measured by absorbance at 450-540 nm using the ClarioStar plate reader (BMG LABTECH).

For starvation conditions the cells were plated at 70% confluence into 96-well plates. After overnight attachment, the media was changed to starvation media (DMEM no glucose, no L-glutamine, no phenol red, no sodium pyruvate) and the cells were treated with βHb 10mM alone or in combination with one of the nutrient components (0.25mM pyruvate, 1mM glucose and 10mM L-glutamine). On the 6th day, the BrdU label was added to each well and incubated for 24 h. The levels of antibody bound to the DNA-incorporated BrdU was detected by analysing the media color change caused by an enzymatic reaction and measured as an absorbance at 450-540 nm using the ClarioStar plate reader (BMG LABTECH).

### Wound healing assay

Cells were seeded in 6-well plates, at 4.8 × 10^5^ cells/well. After 24 h, at nearly 90% confluence, cells were scratched with a 10 µl tip and washed twice with serum-free DMEM media in KD condition or PBS in starvation condition. The appropriate media was added, low glucose DMEM with 0.5% FBS (reduced to prevent cell proliferation) for KD condition, or DMEM phenol-, glucose-, pyruvate-, and L-glutamine-free with 10% FBS was added in starvation conditions. The cells were treated with 3mM or 10mM βHb. Wells were imaged using an inverted microscope and re-imaged at the same coordinates following set time points of incubation. MCF7 and HB2 cells were imaged at 0, 24, and 48 h post wounding, while MDA-MB-231 cells were imaged at 0, 3, 6, 8, 24 h post wounding due to their rapid proliferation. Wound area was measured manually with ImageJ software. The percentage of wound was calculated using the following the formula: final area / initial area × 100%.

### Chemotaxis assay

Cells were seeded as 1000 cells per well in Matrigel 1 mg/ml dissolved in appropriate media no FBS with or without 10mM of βHb into Incucyte® Clearview 96 wells Plate after membrane priming. Following the matrix polymerization the modulator was added to the plate. The upper media contained KD or starvation media with or without βHb while the attractant was the same media with 10% FBS. The number of cells on the bottom membrane was scanned and analyzed every 2 h by the Incucyte® Live-Cell Analysis System.

### Methylene blue

The cells were seeded in 6-well plate as 90% confluence, after overnight attachment the media was changed to starvation media with or without βHb 3 or 10mM for 7 days. The cells to be stained were washed with PBSx1 and methanol with methylene blue [0.5% methylene blue (Cat#M4159, Sigma-Aldrich) dissolved in 50% methanol (Cat#S93301, Thermo-Fisher) and complete volume with ddH_2_O] was added and incubated for 4 h at room temperature. The plates were washed in water and imaged after air-drying.

### Metabolic profiling

MCF7 and MDA-MB-231 cells were cultured in 12-well plate in DMEM complete growth media (15,000 cells/well). After two days, cells were exposed to low glucose DMEM-media with or without 10mM of βHb. The next day (considered as a day 1) for the duration of 5 days, 20ul of cell media were collected from each well to ice cold Eppendorf tubes containing metabolite extraction buffer (50% methanol, 30% acetonitrile and 20% LC-MS-grade water) and vortexed vigorously for 15 s to allow metabolite extraction. Supernatants containing the extracted metabolites were collected after centrifugation at max speed, 4^o^C, for 10 min and stored at -80^o^C until further LC-MS analysis.

### Seahorse assay

MCF7 and MDA-MB-231 were plated at 5000 and 7000 cells per well (respectively) onto a Seahorse XFp Microplate and grown for 24 h. Then, the media was replaced, and three wells were treated with 10mM of βHb for another 24 h. The day before the experiment the media was replaced with XF Real-Time ATP Rate Assay Media, and wells were treated with 10mM of βHb. At least 12 h before the experiment the sensor cartridge was hydrated at 37°C in a non-CO_2_ incubator overnight according the manufacturer instructions . On the day of experiment the cells were washed with XF Real-Time ATP Rate Assay Media and incubated for 60 min in a non-CO_2_ incubator at 37°C. The water in the sensor cartridge was replaced with prewarmed XF Calibrant and the sensor cartridge was incubated for 60 min in a non-CO_2_ incubator. Oligomycin and rotenone + antimycin A were resuspended in the assay media according to as manufacturer protocol in the assay media. These mixes were loaded into the ports of the hydrated sensor cartridge, and the plate entered the Seahorse XF mini machine for XFp Real-Time ATP Rate analysis. Total protein content was assessed immediately after Seahorse measurements using Bradford reagent and standard BSA concentrations. Subsequently, these protein concentrations were entered into the Agilent Seahorse XFp analytic software, which automatically generated graphs with normalized data. Furthermore, ATP production rates were normalized and presented as a percentage of control, where controls for both glycol- and mitoATP were set at 100%.

### ROS detection

Cells were seeded in 96-well black sided, clear bottom plates, at 4-7 × 10^3^ cells/well and were treated with DMEM phenol free, without pyruvate, and L-glutamine, with additional 10% FBS, 1% P/S and 5.5mM glucose with βHb 3 or 10mM. After incubation, ROS levels were measured using “DCFDA/H2DCFDA-Cellullar ROS Assay Kit” (Cat#ab113851; Abcam) according to the manufacturer’s protocol. The DCFDA taken into the cells is deacetylated by cellular esterases to a non-fluorescent compound which is later oxidized by ROS into DCF, which is highly fluorescent and detected by fluorescence at (485_Ex_/535_Em_ nm), using ClarioStar plate reader (BMG LABTECH).

### RNAseq analysis

Cells for RNA sequencing were seeded at 1.2-2 × 10^5^ cells in 100 mm plates in high glucose media and incubated overnight to allow for attachment. After 24 h, the medium was changed to low glucose DMEM (5.5mM; Cat#31,885,023, Gibco), containing βHb at concentrations of 3 or 10mM. Control groups were treated with low glucose media without βHb. The media was replaced every 72 h over the course of 6 days of incubation. RNA of treated and control MCF7, MDA-MB-231, and HB2 cells was extracted using Quick-RNA™ MiniPrep Plus Kit (Zymo; Cat#ZR-R1058, Biological Industries). Quality control for total RNA was performed with TapeStation (Agilent). The RNA integrity number (RIN) of all samples was measured and RNAseq was performed at the Weizmann Institute (Crown Genomics Institute of the Nancy and Stephen Grand Israel National Center for Personalized Medicine, Weizmann Institute of Science). The libraries were prepared using MARSseq protocol and sequenced by Illumina Novaseq sequencer. The yield was 8-40 M reads at a length of 75 bp in single side duplicate reads. The results were evaluated using enrichment analysis IPA (Ingenuity Pathway Analysis, QIAGEN) software, and Galaxy software. The quality control (QC) for all samples was deemed sufficient and the fragments were mapped to GRCh38 genome. The significance was set for genes with p adjustment following Benjamini & Hochberg p.adj < 0.5 and fold change (FC) > 2.

### Quantitative real-time PCR (qPCR)

Aliquots of the same RNA used for RNAseq analysis were reverse transcribed using Revert Aid First Strand cDNA Synthesis Kit (Cat#K1621, Thermo-Fisher). qPCR was carried out in 12 µl triplicates consisting of 6x PCR SYBR Green master mix (AriaMx Real-Time PCR, Santa Clara, USA), 100nM of forward and reverse primers, and 10 ng of cDNA. Gene list and primer sequences are shown in Supplementary Table 1. β-actin was used as a housekeeping gene for endogenous normalization. All genes were detected by qRT-PCR, according to the following profile: 30 s at 95°C, 30 s at 55°C and 30 s at 95°C. Reactions were carried out in the Agilent Aria 1.5 (Santa Clara, USA).

### Statistical analysis

The statistical significance of results in comparable groups was assessed using GraphPad Prism 7 software, San Diego, CA, USA. Independent measurements from separate experiments, representing biological replicates and demonstrating experimental reproducibility, were combined and analyzed using SuperPlots as described by Lord S.J. et al. [27].

## Results

### The effect of the βHb treatment on cell oncogenicity is dependent on the concentration of nutrients

**Fig. 1:**
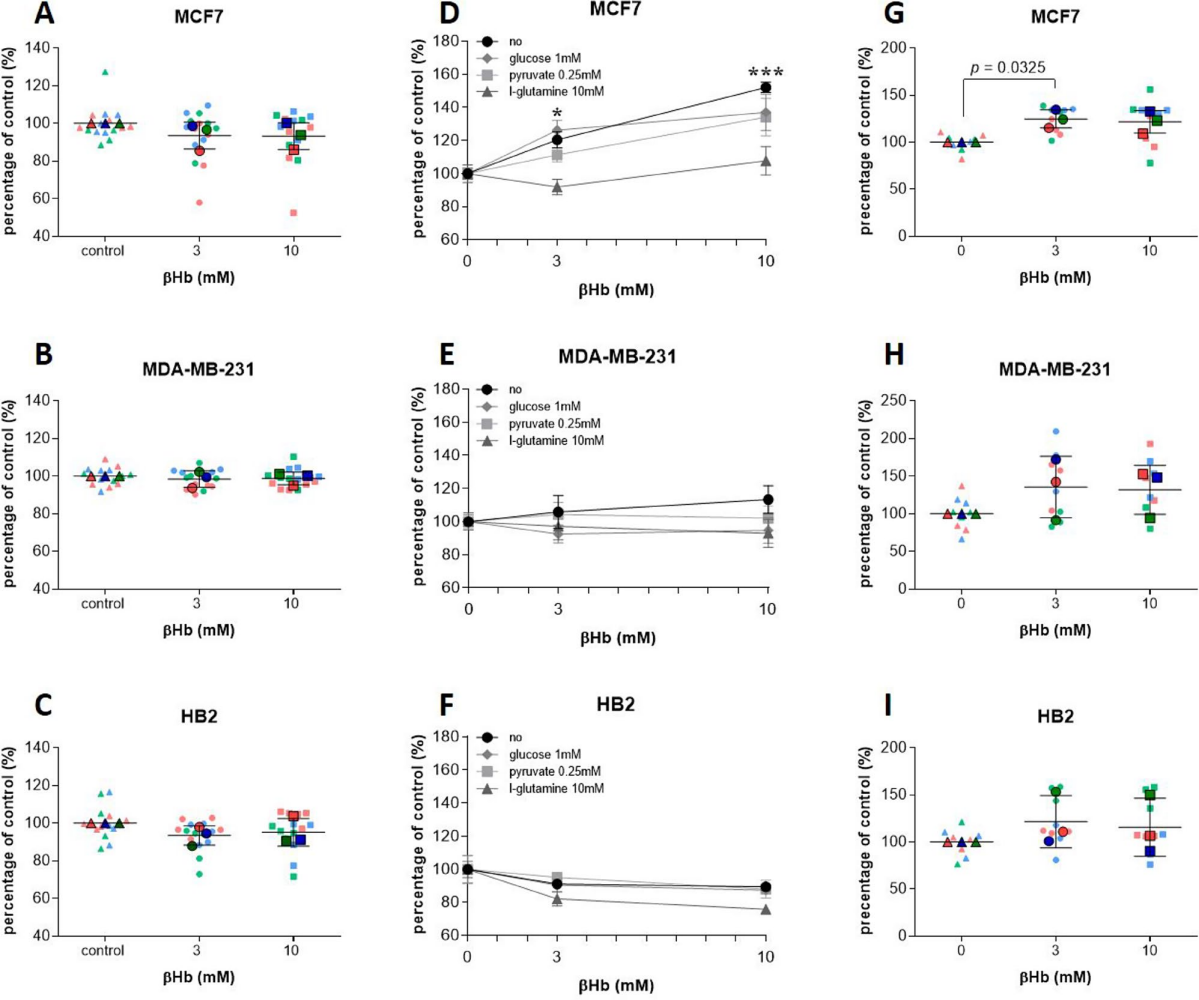
The effect of βHb on cell viability is dependent on the concentration of other nutrient components. (**A-C**) The cytotoxic effect of βHb detected by resazurin assay after 96 h in KD conditions. SuperPlots represents three biological replicates with *n* = 5 for each replicate. (**D-F**) Cells grown for 7 days under starvation with 10mM βHb in combination with pyruvate, glucose, or L-glutamine, or with no nutrients added. The “no” group refers to the starvation media with only 10mM βHb. Data represents three (MCF7 and MDA-MB-231) and two (HB2) biological replicates with *n* = 3 for each replicate. (**G-I**) βHb treatment significantly increases proliferation of MCF7 cells measured by BrdU incorporation assay under KD condition. Cell viability was assessed by resazurin assay measured at 560_Ex_/590_Em_. SuperPlots represents three biological replicates with *n* = 3 for each replicate, and shown as a percentage of control. The significance was determined by two-way (**D**) and one-way (**G**) ANOVA with Tukey post-hoc analysis. Data are presented as mean ± SEM with *** *p* < 0.001

To explore the effects of βHb on the oncogenicity of breast cancer cells, we analyzed cell viability, proliferative, migrative, and invasive abilities. We grew MCF7 and MDA-MB-231 cancer, as well as non-cancer HB2 breast cell lines with two doses (3 or 10mM) of βHb. We mimicked KD conditions by growing the cells for 96 h in low glucose media (5.5mM glucose DMEM, 1mM pyruvate, and 10mM L-glutamine), while starvation conditions were lacking glucose and several other key nutrients in the media (DMEM, no L-glutamine, no sodium pyruvate, with 10% FBS added). Cells were incubated for 7 days with or without βHb. First, we assessed cell survival in control conditions by resazurin assays, which revealed no effect of βHb on the viability of breast cancer nor non-cancer cells (Fig. 1A-C). We found that under KD conditions, βHb did not exhibit any cytotoxic effect.

To assess the interaction between individual nutrients and βHb, we grew cells under varying concentrations of glucose, L-glutamine, and pyruvate, with and without βHb and assessed cell viability by resazurin. We showed that under nutrient starvation conditions treatment with 10mM βHb significantly increased the viability of MCF7 cells (close to 100%) while MDA-MB-231 and HB2 cell survival was not affected by βHb administration (Suppl. Figure 1 A). Interestingly, in MCF7 cells, a low concentration of glucose (1mM) enhanced cell survival in the presence of 10mM βHb while at high concentrations of glucose no significant effect of βHb supplement could be detected. Conversely, the addition of L-glutamine demonstrated a dose-dependent synergistic effect, significantly increasing cell survival in the presence of βHb (10mM) (Suppl. Figure 1 A). MDA-MB-231 and HB2 cell viability showed only slight variability in the presence of selected nutrients (Suppl. Figure 1B-C). These results demonstrate that the effect of βHb supplement depends on the concentration of other nutrients, as well as on the type of cancer cells. Following these results, we utilized the concentrations of nutrients that demonstrated the biggest effect of βHb supplementation (0.25mM pyruvate, 1mM glucose, 10mM L-glutamine) and analyzed dose-dependent effects of βHb alone and in combination with each of the described nutrients. The results confirmed that βHb increases the survival of MCF7 cells in a dose-dependent manner under starvation (Fig. 1D). There was no effect of βHb treatment on MDA-MB-231 (Fig. 1E) and HB2 (Fig. 1F) cell survival.

After observing a significant effect of βHb on the survival of MCF7 cells under starvation, we sought to determine if this effect was indicative of enhanced proliferation in addition to the prevention of cell death. To evaluate the influence of βHb on proliferation, we employed a BrdU incorporation assay under both KD and starvation conditions. We found that in KD conditions, 3 mM of βHb supplementation leads to a significantly higher rate of proliferation in MCF7 cells (Fig. 1G) with a non-significant increase in MDA-MB-231 and HB2 cells (Fig. 1H-I). At the same time, under starvation conditions, βHb alone significantly increases proliferation of MCF7 cells, decreases proliferation rates of HB2 cells, and does not affect MDA-MB-231 cell proliferation (Suppl. Figure 1D-F). Neither the combined treatment of βHb with pyruvate 0.25mM nor with glucose 1mM affected cell proliferation, while a significant increase in proliferation was observed in MDA-MB-231 cells treated with βHb in combination with 10mM L-glutamine (Suppl. Figure 1E).

**Fig. 2:**
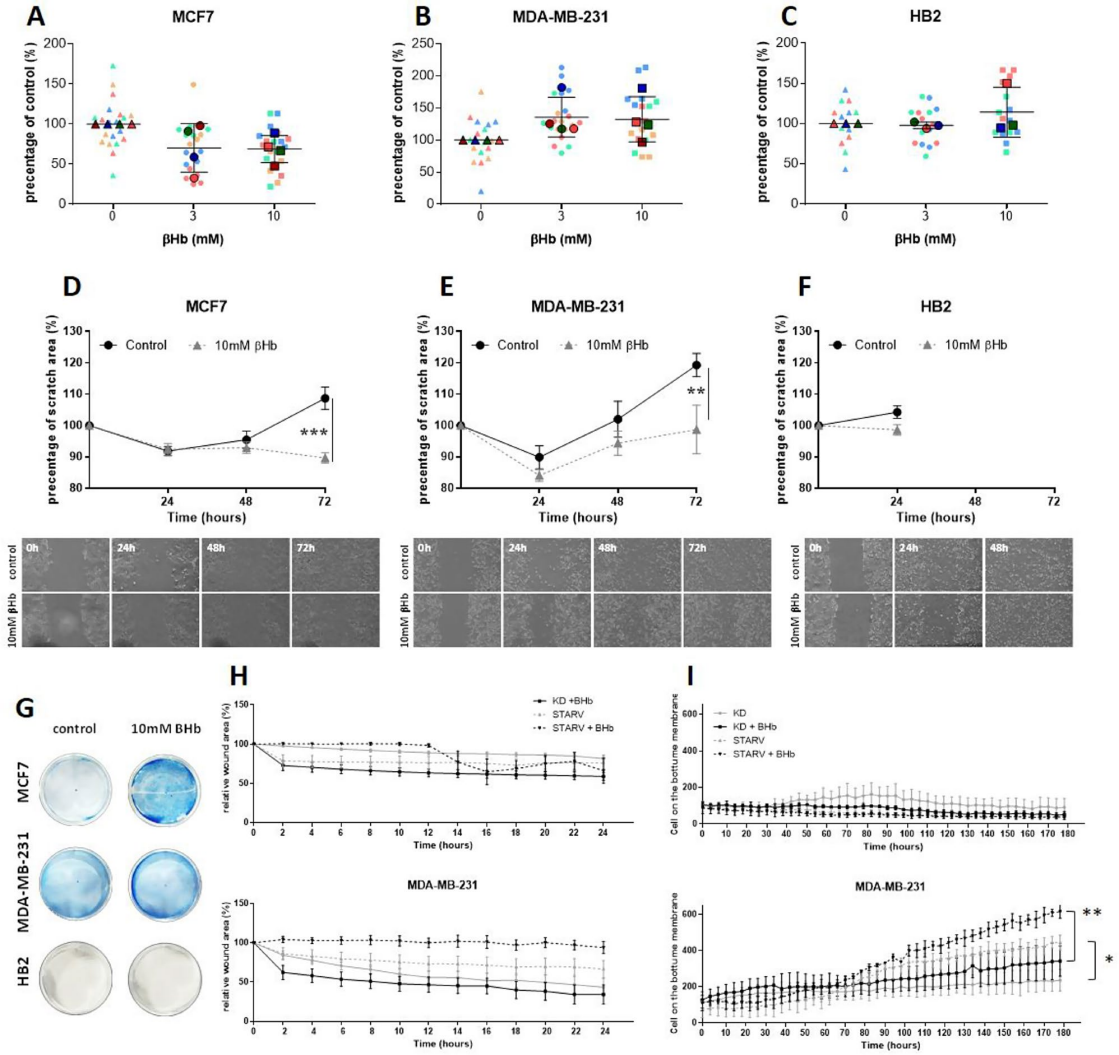
βHb supplementation leads to differing effects on colony formation, survival, and invasion of breast cancer cells (**A-C**) βHb demonstrates an opposite tendency on the colony formaion capabilities of MCF7 and MDA-MB-231 cells . Cells were seeded as single cells and treated with βHb at final concentrations of 3 and 10mM in KD media. Control groups were treated with the same media without βHb. Individual colonies were stained by methylene blue, manually counted, and the percentage of control of colonies was calculated. The significance was determined by one-way ANOVA with Tukey post-hoc analysis. SuperPlots represents four (MCF7 and MDA-MB-231) and three (HB2) biological replicates with *n* = 5 for each replicate, and presented as mean ± SD of the biological replicates average. (**D-F**) βHb treatment significantly affects cell survival under starvation. Cells plated as 4.8 × 10^5^ per well in 6-well plates were treated with 10mM βHb in starvation media. Cells were imaged (10x) at several time points. The graphs represent the average of wound area as a percent of 0 h, the average of two different areas per well of two wells imaged and analyzed. The significance was determined by one-way ANOVA with Tukey post-hoc analysis. Data are presented as mean ± SEM, ** *p* < 0.01, *** *p* < 0.001, *n* = 6 compared with control. (**G**) The plates stained with methylene blue on day 7. (**H**) Cells were treated with 10mM βHb in KD or starvation media for 24 h before the experiment, the wound area was measured automatically by Incucyte®Live-Cell Analysis System. The graphs represent the average wound area as a percent of 0 h for *n* = 10. (**I**) The number of cells in the wound area was detected by Incucyte®Live-Cell Analysis System (*n* = 3). The significance was determined by Two-way ANOVA with Tukey post-hoc analysis. Data are presented as mean ± SEM with * *p* < 0.05, ** *p* < 0.01

As an additional measurement of cell survival, growth, and proliferative capacity we used a colony assay for the cells under KD conditions (Fig. 2A-C). We found that βHb showed a tendency to decrease the ability of MCF7 cells to form colonies (Fig. 2A) while MDA-MB-231 showed the opposite effect (Fig. 2B). HB2 colony formation showed no change in the presence of βHb treatment (Fig. 2C). We also intended to assess the impact of βHb on cell migration capability through a scratch wound assay. Our analysis revealed that the addition of βHb did not influence cell migration in KD conditions (Suppl. Figure 2). However, contrasting results were observed when cells were subjected to starvation (Fig. 2D-G). Due to the cell death in the area surrounding the wound of the control group in MCF7 cells, the wound size increased throughout experiment, whereas the wound size of cells treated with βHb was significantly decreased after 72 h of experiment (Fig. 2D). The same increase of the wound area was observed in MDA-MB-231 control cells, while the death of βHb treated cells was significantly slowed (Fig. 2E, G). Hence, while βHb treatment increased the viability of MCF7 cells after 72 h (Fig. 2D) and slowed the death of βHb treated MDA-MB-231 cells (Fig. 2E) its impact on migration could not be evaluated due to the death of the control cells (Fig. 2D-H). The invasion of MDA-MB-231 cells was also affected by βHb demonstrating significantly enhanced invasion in a chemotaxis assay (Fig. 2I). The experiment was conducted using two setups: one involving KD conditions with and without the addition of 10mM βHb, and the other involving starvation conditions with and without the addition of 10mM βHb. Although no significant differences were observed within the groups, there was a significant distinction between the KD and starvation counterparts (*p* < 0.05), as well as between KD + βHb and Starvation + βHb (*p* < 0.01) conditions in MDA-MB-231 cells from the 100-hour mark after the start of the experiment (Fig. 2I).

### βHb treatment significantly elevates mitochondrial activity of MCF7 cells but not MDA-MB-231 cells

**Fig. 3:**
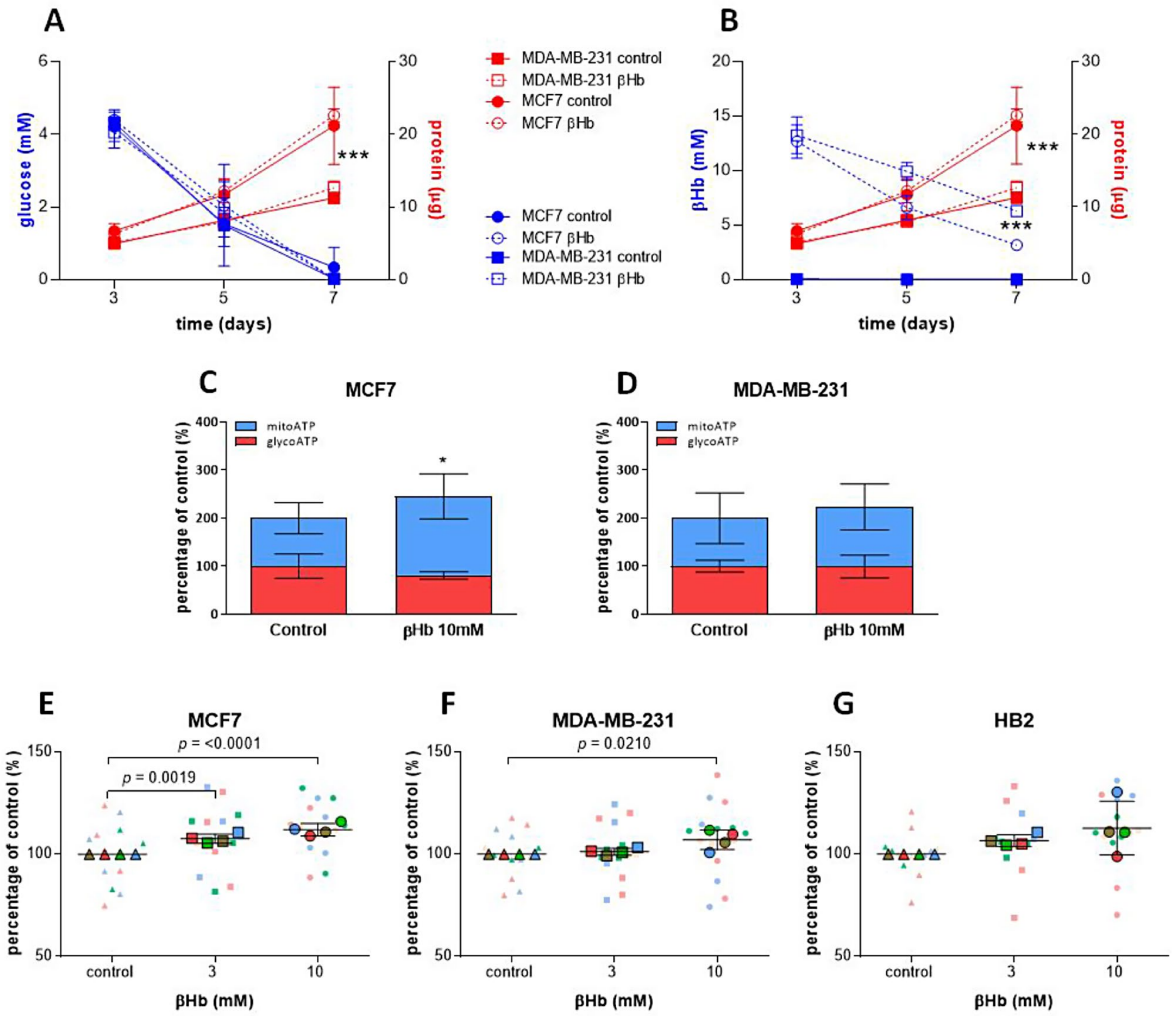
βHb supplementation alters the metabolic source of ATP. (**A-B**) Correlation between cell growth (indicated by protein concentration) and glucose uptake (**A**), as well as βHb consumption (values normalized by sample protein concentration) (**B**) by MCF7 and MDA-MB-231 cells. Data are expressed as mean ± SEM. *** *p* < 0.001, *n* = 3. The significance was determined by two-way ANOVA with Bonferroni post-hoc analysis. (**C-D**) The rates of ATP production in MCF7 and MDA-MB-231 cells in the presence of 10mM βHb measured by SeaHorse technique, and represented as the percentage of control, *n* = 3. (**E-G**) The ROS level measured in MCF7, MDA-MB-231 and HB2 cells in the presence of 3 and 10mM βHb using DCFDA/H2DCFDA-Cellullar ROS assay kit. Results are shown as a percent of control. SuperPlots are expressed as mean ± SD, *n* = 3–4 for four biological replicates. The significance was determined by one-way ANOVA with Tukey post-hoc analysis

To profile the metabolic processes in the cells, we measured glucose uptake and lactate secretion by LC-MS as indicators of glycolysis. We found no significant difference in glucose uptake between the tested cell lines, while cell growth (as determined by protein concentration) was significantly higher in MCF7 cells (Fig. 3A). The presence of βHb did not affect glycolysis showing no changes in glucose uptake, nor lactate production (Fig. 3A, Suppl. Figure 1G). To confirm the utilization of βHb by cancer cells, we examined their βHb consumption. Our results revealed that although both cell lines demonstrated utilization of βHb, the MCF7 cells showed greater βHb consumption compared to MDA-MB-231 cells (Fig. 3B). Next, we sought to investigate whether cancer cells utilize βHb as an alternative energy source through mitochondrial metabolism. We employed the SeaHorse technique to differentiate between ATP produced through glycolysis and ATP produced by the mitochondria. We found mitochondrial ATP to be significantly increased in MCF7 cells in the presence of βHb (Fig. 3C). The oxygen consumption rate (OCR), indicative of mitochondrial respiration, was also significantly higher in MCF7 cells (control: mean = 112.6, SD = 16.48; βHb: mean = 173.89, SD = 35.40) compared to MDA-MB-231 cells (control: mean=-6.86, SD = 7.4; βHb: mean=-5.33, SD = 10.86), demonstrating an increase in mitochondrial activity with βHb 10mM supplementation in MCF7 cells (Suppl. Figure 3A,B). Oligomycin, rotenone, and antimycin A (Rot/AA), which inhibit ATP synthase and complex I of the electron transport chain, led to the downregulation of mitochondrial respiration in MDA-MB-231 cells (control: mean = 18, SD = 2.4; βHb: mean = 17.27, SD = 1.03; Suppl. Figure 3C). Surprisingly, oligomycin triggered an increase in the glycolysis-associated extracellular acidification rate (ECAR) in MCF7 cells, which was further eliminated by the addition of Rot/ AA, while these changes were not linked to βHb administration (control: mean = 36.19, SD = 7.82; βHb: mean = 33.84, SD = 3.99; Suppl. Figure 3D). Increase of mitochondrial ATP production in MCF7 cells was accompanied by a significant increase in ROS levels, further indicating enhanced oxidative phosphorylation (Fig. 3E-G; Suppl. Figure 3E) and, consequently, high mitochondrial activity. MDA-MB-231 cells didn’t present any changes in mitochondrial activity in the presence of βHb (Fig. 3D, F; Suppl. Figure 3 F).

### βHb significantly affects the expression of genes involved in βHb transport and signaling

**Fig. 4:**
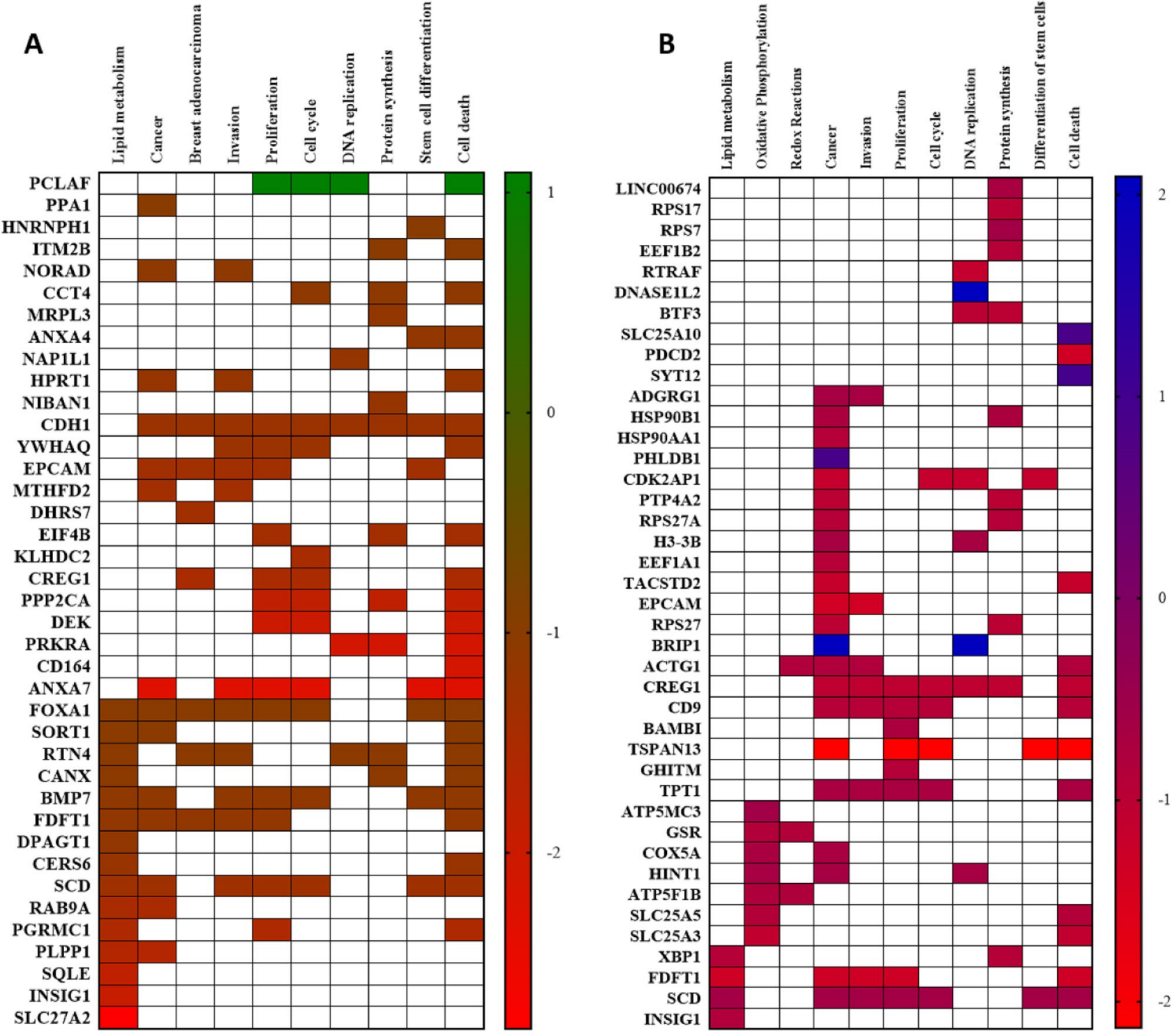
Heatplot analysis based on RNAseq results of MCF7 cells treated with 10mM of βHb. (**A**) Data generated with pathway enrichment analysis using IPA (Ingenuity Pathway Analysis, QIAGEN) software. (**B**) Data generated using usegalaxy.org, a publicly available tool. All presented genes are significantly up or downregulated (p. adj. <0.05) with log2 Fold changes > 2

In order to expand our understanding of the mechanism by which βHb affects breast cancer and non-cancer cell metabolism, we performed RNAseq on cells treated with two doses of βHb (3 and 10mM) in low glucose media (5.5mM glucose DMEM). By conducting pathway enrichment analysis using IPA (Ingenuity Pathway Analysis, QIAGEN) software, we identified a total of 56 significantly downregulated genes and 2 upregulated genes in MCF7 cells treated with 10mM βHb. These genes primarily belong to metabolic pathways, specifically lipid metabolism, and are also associated with pathways related to cancer (Fig. 4A). Furthermore, additional analysis was performed using usegalaxy.org, a publicly available tool, with further adjustment of p-value (< 0.05) and fold change (> 2) criteria. This analysis identified 56 downregulated genes and 7 significantly upregulated genes. Notably, three of these genes (FDFT1, SCD, and INSIG1) were found to overlap with the genes identified by the IPA software. Gene ontology analysis revealed that these genes are also involved in metabolic and cancer pathways (Fig. 4B). Notably, the downregulated genes in this analysis were primarily associated with oxidative phosphorylation signaling (SLC25A3, SLC25A5, ATP5F1B, HINT1, COX5A, GSR, and ATP5MC3), rather than with lipid metabolism (FDFT1, SCD, INSIG1, and XBP1).

**Fig. 5:**
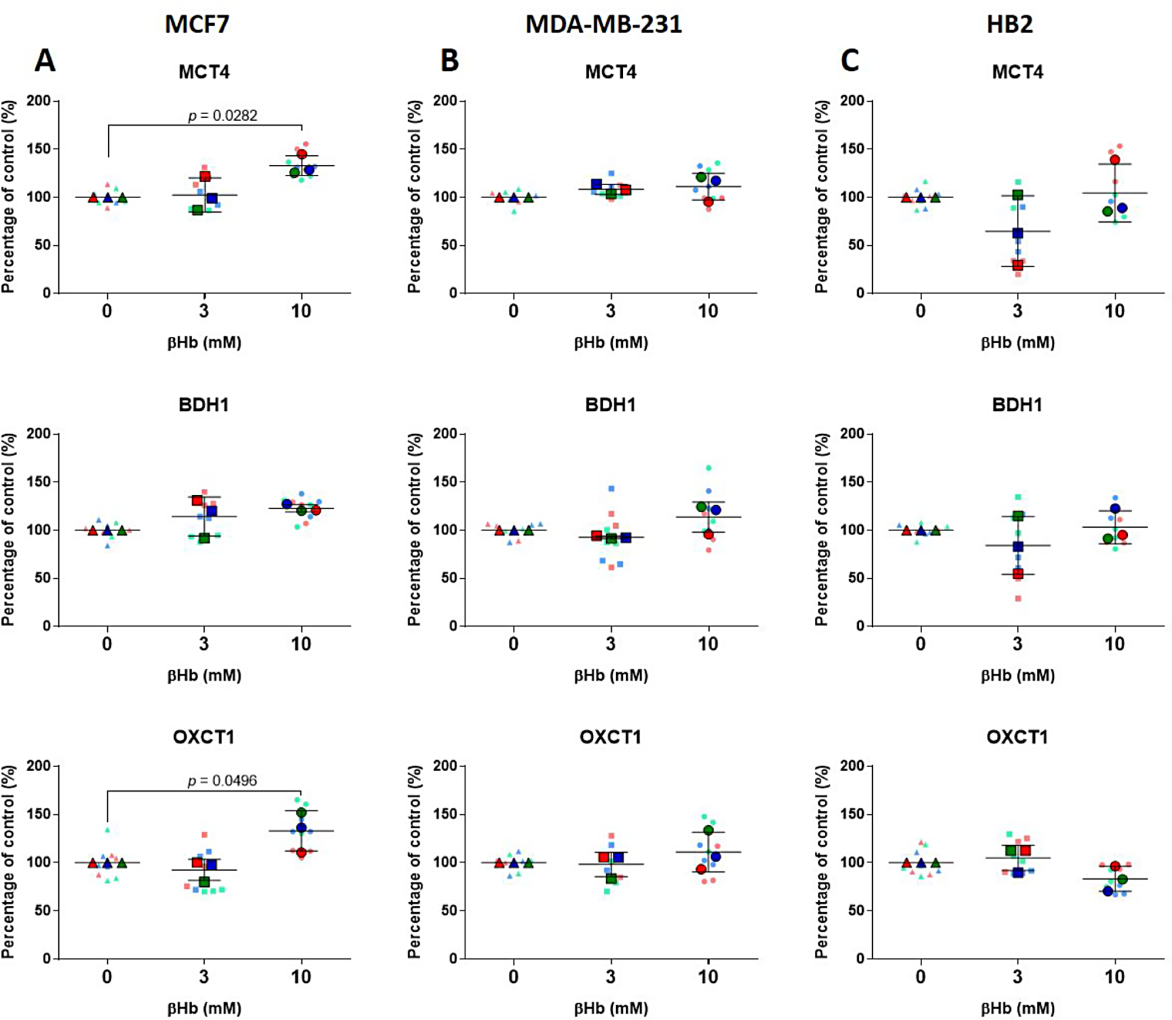
Effect of βHb treatment on MCF7, MDA-MB-231, and HB2 cells gene expression measured by qPCR. (**A-C**) Expression of genes encoding MCT4 transporter and BDH1 and OXCT1 enzymes involved in βHb transport and oxidation in the mitochondria of MCF7 (**A**) MDA-MB-231 (**B**) and HB2 (**C**) cells. The significance was determined by one-way ANOVA with Bonferroni post-hoc analysis. SuperPlots represents three biological replicates with *n* = 3 for each replicate

To validate the RNAseq results, we conducted quantitative real-time PCR (qPCR). Despite the lack of βHb-induced changes in gene expression profiles observed in MDA-MB-231 and HB2 cells according to the RNAseq results, we performed qPCR analysis on the altered genes identified in MCF7 cells for these cell lines as well. First, we analyzed the expression of genes known to be involved in βHb metabolism. Specifically, we focused on the two βHb transporters: monocarboxylate transporter 1 (MCT1) and MCT4 [28, 29]. Our results indicated that the expression of the membrane MCT1 transporter was not significantly altered. However, the membrane and mitochondrial transporter MCT4 was significantly upregulated in MCF7 cells when treated with 10mM βHb, while its expression remained unaffected in MDA-MB-231 and HB2 cell lines (Fig. 5A-C). Further, one of the two enzymes involved in βHb oxidation in the mitochondria [30] 3-hydroxybutyrate dehydrogenase 1 (BDH1) showed tendency to upregulation whereas 3-oxoacid CoA-transferase 1 (OXCT1), was significantly increased in MCF7 cells (Fig. 5A). Additionally, treatment of MCF7 cells with 10mM βHb resulted in a significant downregulation of AMP-activated protein kinase (AMPK) and a dose-dependent tendency to upregulation of HDAC1, both known to be associated with βHb metabolism. However, the expression of FOXO3a, the transcription factor regulated by AMPK [31], was not affected (Suppl. Figure 4).

Further, we conducted qPCR analysis on selected genes involved in the lipid metabolism pathway that exhibited significant changes: Slc27a2, INSIG1, SQLE, SCD1, CerS6, FDFT1, FOXA1, GPAGT1, PLPP1, and RAB9A. qPCR analysis confirmed a significant downregulation of SQLE, SCD1, FDFT1, FOXA1, and PLPP1 genes in MCF7 cells treated with 3mM βHb. However, changes in Slc27a2, RAB9A, and GPAGT1 gene expression were not replicated (Suppl. Figure 4). Interestingly, in contrast to MCF7 cells, INSIG1, PLPP1, and RAB9A and genes were significantly upregulated in MDA-MB-231 cells treated with 10mM βHb. The gene expression pattern of HB2 cells, analyzed by qPCR, was similar to that of MCF7 cells. Consequently, SQLE, SCD1, and PLPP1 genes exhibited significant downregulation in HB2 cells. Additionally, two genes (Slc27a2 and CerS6) were significantly downregulated by both doses of βHb in HB2 cells only (Suppl. Figure 4). These findings suggest that βHb affects lipid metabolism differently in the two cancer cell lines.

We also examined the effect of βHb on the expression of Glut1, a marker of glycolysis [32], but no significant changes were observed. Interestingly, the signal transducer and activator of transcription 1 (STAT1), known to regulate genes involved in glycolysis [33], showed significant downregulation in MDA-MB-231cell line following βHb treatment, while it was upregulated in non-cancerous breast cells. STAT2 expression was downregulated in MCF7 cell only with no effect on MDA-MB-231 and HB2 cells. Finally, we found that hypoxanthine guanine phosphoribosyltransferase (HPRT), known to control pyrimidine synthesis in response to energy abundance [34] and commonly overexpressed in malignancy, was upregulated in MCF7 cells following βHb treatment (Suppl. Figure 4).

## Discussion

Cancer cells are characterized by their rapid growth and altered metabolic pathways, which involve increased glycolysis and reduced oxidative phosphorylation [9]. Ketogenic diet (KD), which is high in fat and low in carbohydrates, has been studied for its potential benefits in various diseases, including cancer [9]. When following a KD, the circulating levels of ketone bodies (KB) are elevated, providing an alternative energy source [3]. Thus, the main objective of this study was to examine the impact of KD on breast cancer using an in vitro model. To simulate KD conditions, we cultured the cancer cell lines MCF7 and MDA-MB-231, along with the non-cancer cell line HB2, in low-glucose media supplemented with β-hydroxybutyrate (βHb).

Our findings demonstrated that MCF7 cells exhibited a greater sensitivity to βHb supplementation compared to MDA-MB-231 cells (Figs. 1, 2 and 3). This discrepancy in cellular response may be attributed to previously published data indicating that MDA-MB-231 cells rely more on glycolysis for their energy needs, while MCF7 cells tend to rely more on mitochondrial oxidation [35]. It is also known that MCF7 cells have a greater ability to utilize βHb, which has been associated with an increase in stemness [25, 36]. On the other hand, MDA-MB-231 cells demonstrate enhanced survival and metastatic potential only when ketone oxidation enzymes, such as OXCT1, are overexpressed [36].

We also observed that the addition of pyruvate and glucose, but not L-glutamine, effectively neutralized the survival effects of βHb. This suggests a shift in metabolism towards glycolysis. Conversely, under starvation conditions, the presence of L-glutamine intensified the impact of βHb treatment in MCF7 cells (Suppl. Figure 1 A). This can be attributed to the well-established preference of cancer cells to rapidly consume glutamine, which can provide the metabolic energy, carbon, and amino-nitrogen necessary for nucleotide, lipid, and protein biosynthesis when glucose is limited [37].

We further found that βHb supplementation not only significantly enhanced the survival of MCF7 cells (Figs. 1D and 2D) but also stimulated their proliferation (Fig. 1G). This is notable as previous literature reports have not shown a significant effect of βHb on breast cancer cell proliferation [38]. While normal cells display density-dependent inhibition of proliferation, entering quiescence at maximal concentration, cancer cells show no density-dependent inhibition and continue growing even at high cell densities [39]. This might be due to their reprogrammed metabolism and the microenvironment, which allows cancer cells to continue to proliferate. The discrepancy between proliferation assays and colony-forming assays in the context of cancer cells highlights the importance of cell density and microenvironment [40, 41]. Proliferation assays focus solely on measuring the rate of cell division. Colony-forming assays, however, provide insight into the survival and reproductive capacity of cancer cells under specific density and environmental conditions, shedding light on their adaptive mechanisms and growth potential [42]. Therefore, the BrdU incorporation assay demonstrated a selective increase in proliferation exclusively in MCF7 cells, with no impact on the proliferation of MDA-MB-231 and HB2 cells (Fig. 1D-I). The colony-forming assay demonstrated another effect of βHb on cell proliferation, with a tendency to decrease the colony formation capabilities observed in MCF7 cells and to increase it in MDA-MB-231 cells (Fig. 2A-B). Furthermore, βHb enhanced the invasive abilities of MDA-MB-231 cells (Fig. 2I), with no significant effect observed in the other cell counterparts. The differential effects of βHb treatment on various cell lines may be explained by their distinct preferences and consumption of other nutrients present in the FBS-enriched media. Therefore, we may speculate that the effect of βHb depends on the specific nutrient composition of the cellular environment. These contradictory results further underscore the controversial impact of βHb on oncogenicity [24, 36, 43], which also may be associated with elevated aggressiveness of MDA-MB-231 cells, linked to the lack of expression of estrogen receptor (ER), progesterone receptor (PR), and human epidermal growth factor receptor 2 (HER2), as well as with the expression of ER and PR in the absence of HER2 in MCF7 cells [44, 45]. Further work is necessary to understand if these trends are correlated with breast cancer subtype or to some other molecular characteristics. Our hypothesis regarding the distinct preferences and consumption of different nutrients by various cell lines was further supported by the analysis of βHb consumption and utilization, as well as measurements of glucose uptake, lactate secretion, and reactive oxygen species (ROS) production, which serve as indicators of changes in the balance between glycolysis and oxidative phosphorylation. Despite the lack of detectable changes in glucose consumption or lactate production under KD conditions which was mimicked by βHb treatment (Fig. 3A-B; Suppl. Figure 1G; [24]), we observed differential consumption and production of these nutrients between MCF7 and MDA-MB-231 cells.

The response of cancer cells to elevated levels of ROS depends on the concentration and localization of ROS within the cell, and it can have different effects on carcinogenicity, including proliferation, migration, and cell survival [46]. MCF7 cells have been observed to exhibit increased survival under prolonged exposure to ROS [47], which may explain the correlation we observed between high ROS levels and enhanced survival and proliferation of MCF7 cells in our experiments (Fig. 3E-G). Additionally, MCF7 cells are known to be more sensitive to ROS elevation in response to L-glutamine depletion compared to MDA-MB-231 cells [48]. In line with this, we found that the addition of L-glutamine under starvation conditions increased the effect of βHb on the viability of MCF7 cells (Suppl. Figure 1D). Moreover, cells can convert L-glutamine into glutathione (GSH by glutathione synthetase) [49], an antioxidant utilized by cells to moderate oxidative stress caused by ROS [49]. Interestingly, our RNAseq results revealed a significant downregulation of glutathione-disulfide reductase (GSR), the enzyme responsible for reducing oxidized glutathione disulfide (GSSG) to its sulfhydryl form GSH, in MCF7 cells following βHb treatment (Fig. 4B). This downregulation of GSR may contribute to the increased ROS levels observed [46].

We did not observe any changes in mRNA expression of the glucose transporter Glut1, which is commonly used as a marker for the Warburg effect [32]. However, we found that signal transducer and activator of transcription 1 (STAT1), a known regulator of genes involved in glycolysis [33], was downregulated by βHb specifically in cancer cells and upregulated in HB2 cells (Suppl. Figure 4). Additionally, our gene expression profiling of MCF7 cells (Fig. 5A) showed tendency to upregulation of 3-hydroxybutyrate dehydrogenase 1 (BDH1) and significant elevation of 3-oxoacid CoA-transferase 1 (OXCT1), two enzymes involved in the intramitochondrial oxidation of βHb to acetoacetate (AcA) and acetoacetyl-CoA (AcA-CoA) [30], respectively. These changes in gene expression indicate the activation of βHb metabolism, characterized by an increase in oxidative phosphorylation, which was also confirmed by the elevated levels of ROS observed in cells treated with βHb (Fig. 3E-G).

Another pathway involved in oxidative stress resistance is mediated by the FOXO3a gene, known to be upregulated by acetylation through HDAC1 inhibition under βHb treatment. FOXO3a is a transcription factor regulated by nutrient sensing AMP-activated protein kinase (AMPK) in response to oxidative stress. The AMPK/FOXO3a axis is involved in energy metabolism and promotes cell survival [31]. In our study, βHb supplementation led to downregulation of AMPK, preventing alteration of FOXO3a in MCF7 cells (Suppl. Figure 4)and resulting in high ROS levels (Fig. 3E-G). Indirect downregulation of AMPK was accompanied by the mentioned above increase in OXCT1 expression, known to be involved in AMPK signaling inhibition [50], βHb oxidation [30], as well as increasing MCF7 cell proliferation [51]. Although our findings contradict the conventional view of βHb as an HDAC1 inhibitor [52], analysis of clinical studies using the cBioportal database revealed a highly positive correlation between alterations in the OXCT1 gene and HDAC11 expression. This correlation suggests that when OXCT1 expression is altered by βHb treatment, it may be responsible for the increase in HDAC1 levels.

As previously mentioned, βHb undergoes oxidation in the mitochondria, catalyzed by BDH1, to produce acetoacetate. Acetoacetate is further converted to acetoacetyl CoA by OXCT1 and then transformed into two molecules of acetyl CoA by acetyl-CoA acetyltransferase (ACAT1) [30]. In our study, βHb treatment of MCF7 cells led to significant downregulation of genes involved in endogenous acetyl CoA production (SLC27A2, PLPP1, PGRMC1, SCD, CERS6, DPAGT1, FDFT1, and BMP7) as well as genes related to cholesterol metabolism (INSIG1, SQLE, FDFT1, RTN4, and SORT1; Fig. 4A). Concurrently, OXCT1, and monocarboxylate transporter 4 (MCT4) were upregulated (Fig. 5A). These findings suggest that the external supply of βHb makes it a preferred metabolite as a source of acetyl CoA in these cells. This suggestion is further supported by the understanding that tumor proliferation, survival, metastatic migration, and invasion, especially in breast cancer, are closely linked to lipid metabolism in general and cholesterol synthesis in particular [53].

Finally, among the genes affected by βHb treatment, seven were found to be influenced by insulin activity, which is known to be associated with and regulated by lipid metabolism under KD. One of these genes is insulin-induced gene 1 protein (INSIG1), which is regulated by insulin and plays a role in blocking cholesterol biosynthesis by interacting with the SCAP-SREBP complex was found to be correlated with breast cancer cell viability [54]. We found that βHb treatment in MCF7 cells leads to the downregulation of INSIG1 expression (Fig. 4A). This downregulation results in increased SREBP-coordinated lipid biosynthesis, which in turn promotes cell migration and invasion [55].

## Conclusions

Based on our results, we can summarize that βHb demonstrates non-cytotoxic properties, instead promoting the survival and proliferation of MCF7 cells, while influencing lipid metabolism, potentially serving as an alternative source of acetyl CoA for energy production. In contrast, MDA-MB-231 cells, while displaying a similar inclination to MCF7 cells, exhibit a less pronounced reliance on βHb consumption, with non-significant effects observed in most cases. At the same time, prior studies have indicated the potential benefits of a ketogenic diet for cancer patients [9, 56]. Hence, we may speculate that the differences in the efficacy of βHb treatment depend on the presence and concentration of other nutrients, as well as other inherent characteristics of the cancer cells. We believe that the differential response of cancer cell lines to ketone bodies emphasizes the importance of individual metabolic profile analysis when considering ketogenic diet as an adjuvant treatment for cancer patients.

### Limitations

Due to the known rapid degradation of glutamine and since in the experimental setup, our cells experienced a significant decrease in glucose supply, and given the dual role of glutamine in fueling the TCA cycle and supporting antioxidant mechanisms, we adjusted L-glutamine to 10mM to maintain adequate levels for cellular metabolism. While our approach aligns with established practices, the departure from physiological conditions should be considered when interpreting the results.

### Electronic supplementary material

Below is the link to the electronic supplementary material.

Supplementary Material 1

## Data Availability

All data are provided as figures and tables and included in this paper.

## Abbreviations

KD: ketogenic diet
KB: ketone bodies
βHb: β–hydroxybutyrate
ROS: reactive oxygen species
LC-MS: Liquid chromatography–mass spectrometry
