# Supplementary material for: Metabolic alterations and cellular responses to β-Hydroxybutyrate treatment in breast cancer cells
Source: Cancer Metab. 2024 May 29;12:16. doi: 10.1186/s40170-024-00339-1 (PMC11134656; doi:10.1186/s40170-024-00339-1)
Supplement: Supplementary file 1 — Supplementary Material 1 [file 40170_2024_339_MOESM1_ESM.docx]

**Supplementary materials:**

**Supplementary Figure 1. The effect of βHb in combination with other nutrients on cell viability and lactate production.** (**A-C**) The cytotoxic effect of βHb detected by resazurin assay after 96h in KD conditions. Cells were grown for 7 days under starvation conditions with 10mM βHb in combination with pyruvate, glucose, or L-glutamine, or with no nutrients added. The “low” group refers to the low glucose media with 5.5mM glucose, 1mM pyruvate and 10mM L-glutamine. The “no” group refers to the starvation media with only 10mM βHb. Cell viability was assessed by resazurin assay measured at 560_Ex_/590_Em_. Results are shown as a percentage of control for each nutrient. The significance was determined by one-way ANOVA with Tukey post-hoc analysis. Data are presented as mean ± SEM with *** p<0.001. (**D-F**) βHb treatment selectively affects proliferation under starvation conditions in combination with each of the nutrient components (0.25mM pyruvate, 1mM glucose and 10mM L-glutamine). On the 6th day, the BrdU label was added to each well and incubated for 24 hours. Results are shown as a percentage of control. The significance was determined by one-way ANOVA with Tukey post-hoc analysis. Data are presented as mean ± SEM with * p<0.05, ** p<0.01 *** p<0.001. (**G**) Lactate production by MCF7 and MDA-MB-231 cells measured by LC-MS. Data are expressed as mean ± SEM. ** p<0.01. The significance was determined by two-way ANOVA with Bonferroni post-hoc analysis.

**Supplementary Figure 2. βHb does not significantly affect cell migration under KD conditions.** (**A-C**) Representative images of the wound area (10x magnification). (**D-F**) Average wound area as a percentage of 0hr, the average of two different areas per well of two wells imaged and analyzed. Data are presented as mean ± SEM.


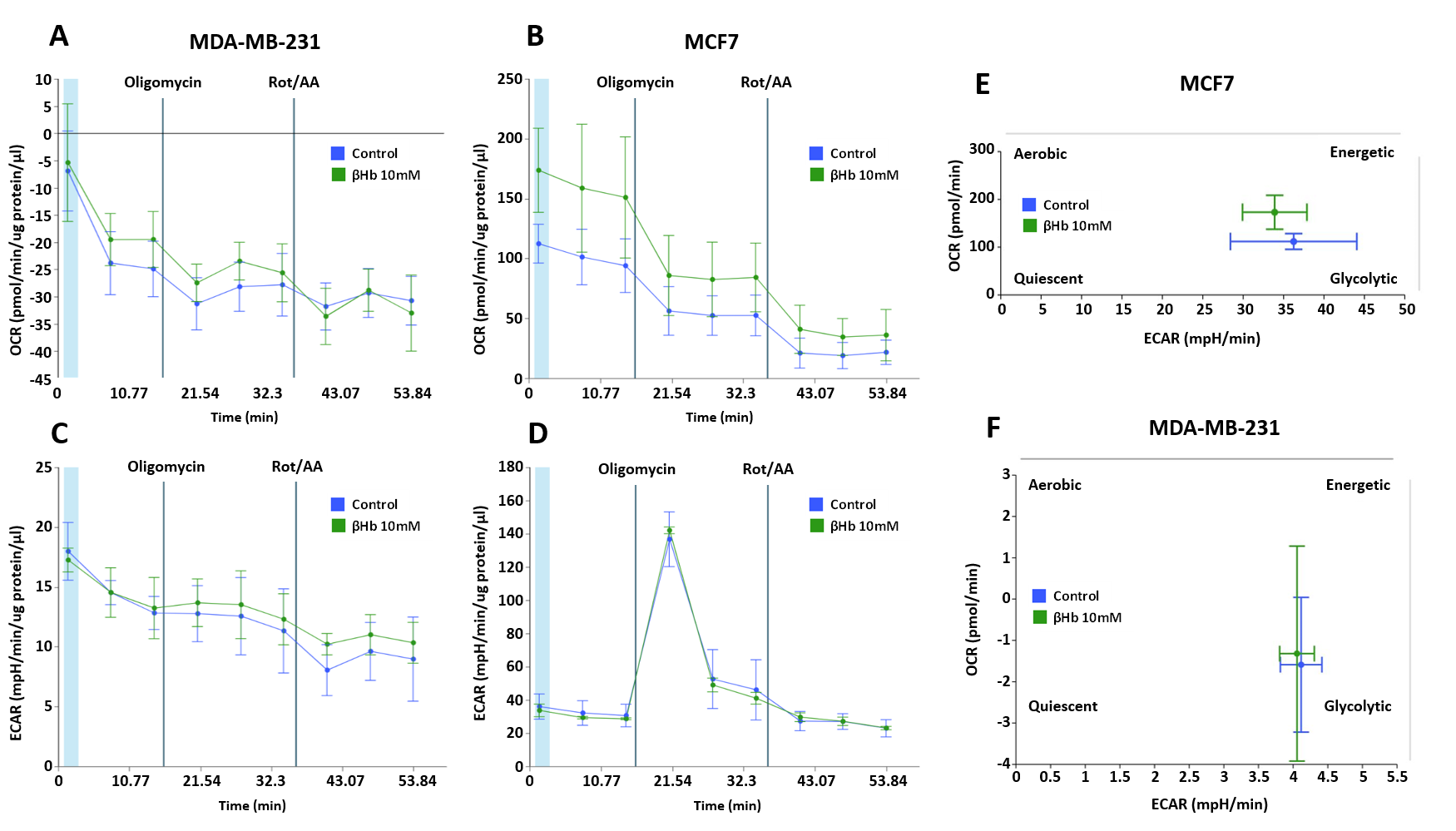


**Supplementary Figure 3. The effect of βHb supplementation on mitochondrial respiration and glycolysis dynamics in MCF7 and MDA-MB-231 cells.** SeaHorse analysis of MCF7 and MDA-MB-231 live cells simultaneously measured 48h after administration with βHb 10mM. **(A, B)** The oxygen consumption (OCR), (**E, F**) extracellular acidification (ECAR), and (**C, D**) energy production rates. Oligomycin, rotenone and antimycin A (Rot/AA) were used to inhibit ATP synthase and complex I of the electron transport chain.


**Supplementary Figure 4. Effect of βHb treatment on MCF7, MDA-MB-231, and HB2 cells gene expression measured by qPCR.** MCF7 (**A**) MDA-MB-231 (**B**) and HB2 (**C**) cells. The significance was determined by one-way ANOVA with Bonferroni post-hoc analysis. SuperPlots represents three biological replicates with n=3 for each replicate.

**Supplementary Table 1. The list of primers used in qPCR**

| **Gene name** | | **Primer Forward** | **Primer Reverse** |
| --- | --- | --- | --- |
| slc27a2 | Solute Carrier Family 27 Member 2 | tttcagccagccagttttg | tctcctcgtaagccatttcc |
| INSIG1 | Insulin Induced Gene 1 | ggcagcttcccaagtattcg | ctacctcctttgggcactga |
| SQLE | Squalene Epoxidase | ttagaggagaaatgccaaggaa | cactgatgaaggaggaaggaag |
| SCD1 | Stearoyl-CoA Desaturase 1 | ctccactgctggacatgaga | aatgagtgaaggggcacaac |
| CERS6 | Ceramide Synthase 6 | tgccattctggaaaaggtct | atgcttcgaacatcccagtc |
| FDFT1 | Farnesyl-Diphosphate Farnesyltransferase 1 | gcaccacatcccagatgtca | ttccgaatcttcactgcccc |
| FOXA1 | Forkhead Box A1 | gcctgagttcatgttgctga | aaaacgcgtattggaactgc |
| DPAGT1 | Dolichyl-PhosphateN-Acetylglucosaminephosphotransferase 1 | gggcgtttcttgccctctac | cctggcccaagttctatccc |
| RAB9A | Member RAS Oncogene Family | ctagtgccacggttgagaaa | cccagccactcactctttaat |
| MCT1 | Solute Carrier Family 16 Member 1 | ttgagccgacctaaaagtggt | tctgtgtctatgcgggattctt |
| MCT4 | Solute Carrier Family 16 Member 3 | cgagtctgcaggaggcttgtg | attggcctggtgctgctgatg |
| GLUT1 | Solute Carrier Family 2 Member 1 | acagcgttgatgccagaca | gatgatgcgggagaagaaggt |
| BDH | 3-Hydroxybutyrate Dehydrogenase 1 | agcaatagtgggcgtcttgc | ggccagtcagcctggaaatct |
| HDAC1 | Histone Deacetylase 1 | cacacttggcgtgtcctttg | gttctgtggcaagtgctgtg |
| OXCT1 | 3-Oxoacid CoA-Transferase 1 | ctctaccactgtggtttctgcag | gctttggtgaaagcctggaagg |
| AMPKa1 | Protein Kinase AMP-Activated Catalytic Subunit Alpha 1 | tgtgacttccaggtcttggagtt | tgcgtgtacgaaggaagaatcc |
| FOXO3A | Forkhead Box O3 | ggactcactcaagcccatgttg | tcaatcagaacttgctccacca |
| STAT1 | Signal transducer and activator of transcription 1 | ggaaggggccatccacattca | gtagggttcaaaccgcatgga |
| STAT2 | Signal transducer and activator of transcription 2 | tcgaaacacctgtggaggagagc | tctgatgggggtccagagag |
